# IL-17C Mitigates Murine Acute Graft-vs.-Host Disease by Promoting Intestinal Barrier Functions and Treg Differentiation

**DOI:** 10.3389/fimmu.2018.02724

**Published:** 2018-11-26

**Authors:** Huanle Gong, Shoubao Ma, Shuangzhu Liu, Yonghao Liu, Ziqi Jin, Ying Zhu, Yuan Song, Lei Lei, Bo Hu, Yu Mei, Hong Liu, Yuejun Liu, Yan Wu, Chen Dong, Yang Xu, Depei Wu, Haiyan Liu

**Affiliations:** ^1^Institute of Blood and Marrow Transplantation, Medical College of Soochow University, Soochow University, Collaborative Innovation Center of Hematology, The First Affiliated Hospital of Soochow University, Suzhou, China; ^2^Immunology Programme, Life Sciences Institute and Department of Microbiology and Immunology, National University of Singapore, Singapore, Singapore; ^3^School of Radiation Medicine and Protection School for Radiological and Interdisciplinary Science, Soochow University, Suzhou, China; ^4^Institute for Immunology and School of Medicine, Tsinghua University, Beijing, China

**Keywords:** IL-17C, Acute graft-vs.-host disease, Treg cells, intestinal barrier functions, inflammation, transplantation

## Abstract

Acute graft-vs.-host disease (aGVHD) is one of the major complications and results in high mortality after allogeneic hematopoietic stem cell transplantation (allo-HSCT). IL-17C is involved in many inflammatory immune disorders. However, the role of IL-17C in aGVHD remains unknown. Here we demonstrated that IL-17C deficiency in the graft significantly promoted alloreactive T cell responses and induced aggravated aGVHD compared with wildtype donors in a fully MHC-mismatched allo-HSCT model. In contrast, IL-17C overexpression ameliorated aGVHD. IL-17C deficiency increased intestinal epithelial permeability and elevated inflammatory cytokine production, leading to an enhanced aGVHD progression. Tregs was reduced in recipients of IL-17C^−/−^ graft, whilst restored after IL-17C overexpression. Decreased Treg differentiation was abrogated after neutralizing IFN-γ, but not IL-6. Moreover, depletion of Tregs diminished the protective effect of IL-17C. Of note, patients with low IL-17C expression displayed higher aGVHD incidence together with poor overall survival, thereby IL-17C could be an independent risk factor for aGVHD development. Our results are the first demonstrating the protective role of IL-17C in aGVHD by promoting intestinal barrier functions and Treg differentiation in a MHC fully mismatched murine aGVHD model. IL-17C may serve as a novel biomarker and potential therapeutic target for aGVHD.

## Introduction

Allogeneic hematopoietic stem cell transplantation (allo-HSCT) is a curative therapeutic strategy for treating malignant hematological diseases. However, acute graft-vs.-host disease (aGVHD) is one of the major complications of allo-HSCT, limiting its clinical application, and prognosis (1). Occurrence of aGVHD is predominantly induced by activation of donor-derived T cells and the production of proinflammatory cytokines, resulting in the damage of host organs such as liver, lung, gut, and skin (2). Presence of inflammatory milieu is critical for the initiation of aGVHD and amplification of alloreactive T cell responses (3, 4). Proinflammatory cytokines including IL-1, IL-6, IFN-γ, and TNF-α contribute to the inflammatory settings and substantially promote the pathogenesis of aGVHD (5–7). Therefore, developing effective therapies to mitigate inflammation is vital in prevention and treatment of aGVHD.

IL-17 cytokine family consists of IL-17A, IL-17B, IL-17C, IL-17D, IL-17E, and IL-17F, and its receptor family includes IL-17RA, IL-17RB, IL-17RC, IL-17RD, and IL-17RE. The role of IL-17A in aGVHD has been well established. IL-17A can be produced by Th17 cells, Tc17 cells, γδ T cells, natural killer (NK) cells, natural killer T (NKT) cells, macrophages, T follicular helper cells, and lymph tissue inducer (LTi) cells (8–14). Studies by our group and others demonstrated that IL-17A could alleviate aGVHD by suppressing Th1 responses and macrophage infiltration (15, 16). Recipient-derived IL-17A has recently been reported in the prevention of dysbiosis and intestinal aGVHD (17). However, Th17 cells augment aGVHD by enhancing IFN-γ production (18–20). Tc17 cells have also been found to promote aGVHD in response to IL-6 and host dendritic cells, without contributing to GVL effects (21). Genetic depletion of IL-17RB in host, which blocks IL-17B and IL-17E signaling, didn't alter the aGVHD-related survival (17). The functions of other IL-17 family cytokines, including IL-17C during aGVHD is still unknown.

Recently, IL-17RE has been characterized as the functional receptor for IL-17C, and IL-17C plays a critical role in the regulation of host defense against infections and autoimmune disorders (22–24). IL-17C was initially demonstrated to stimulate TNF-α and IL-1β production in human THP-1 cells (25). IL-17C can also promote Th17 response in experimental autoimmune encephalomyelitis (EAE) to aggravate inflammation (22). In autoimmune hepatitis, IL-17C augments T cells function by enhancing IL-2 production (26). During *C. albicans* infection, IL-17C amplifies proinflammatory cytokine expression to promote lethal inflammation (27). Thus, IL-17C can regulate T cell responses and inflammatory milieu, which indicates a possible role in aGVHD.

In this study, we first investigated the role of IL-17C in aGVHD in a murine fully-mismatched allo-HSCT model using IL-17C-deficient mice and IL-17C overexpression model. IL-17C could mitigate aGVHD by promoting intestinal epithelia barrier function and Treg differentiation. We further investigated the role of IL-17C in allo-HSCT patients. IL-17C serum levels in more severe aGVHD patients were significantly lower than those with no or moderate aGVHD. In addition, low IL-17C serum level is an independent risk factor for predicting grade II-IV aGVHD. Therefore, IL-17C plays a critical role in aGVHD and could serve as a prognosis marker or therapeutic target in clinical aGVHD management.

## Materials and methods

### Animals

Female BALB/C(H-2^d^), C57BL/6(H-2^b^)and B6D2F1 (F1 hybrid of B6 and DBA/2; H-2^b/d^) mice were purchased from Shanghai Laboratory Animal Center (Shanghai, China). C57BL/6 IL-17C^−/−^ mice were kindly provided by Dr. Chen Dong (Tsinghua University, Beijing, China). C57BL/6 FoxP3-eGFP mice (CD45.2; H-2^b^) were provided by Dr. Zhinan Yin (Jinan University, Guangzhou, China). C57BL/6 CD45.1 mice (H-2^b^) were obtained from Beijing Vital River Laboratory Animal Technology Co. Ltd (Beijing, China). All mice were housed in a specific-pathogen-free environment and received acidified autoclaved water at Animal Facilities of Soochow University. All animal experiments were performed in accordance with the guidelines and approved by the Animal Care and Use Committee of Soochow University.

### Establishment of aGVHD model and histology assessment

Murine aGVHD model was established as previously described (16, 28). Briefly, BALB/C recipients received lethal irradiation of 650cGy (Co60 or X-Ray, 325cGy per dose with 4 h interval) and were injected intravenously with 1 × 10^∧^7 bone marrow (BM) cells and 5 × 10^∧^6 splenocytes (SP) from C57BL/6 or IL-17C^−/−^ mice, respectively. For IL-17C overexpression aGVHD model, BALB/C recipients were injected with IL-17C-expresssing plasmid or vector control (60 ug/2 ml) by hydrodynamic gene transfer 3 days before transplantation. Recipients were conditioned with total body irradiation of 650 cGy by X-Ray in two divided dose 4 h apart (100 cGy/min). BALB/C recipients were transplanted with 1 × 10^∧^7 bone marrow (BM) cells and 5 × 10^∧^6 splenocytes from IL-17C^−/−^ donors. In some experiments, recipients were injected with 0.5 ug/200 ul rmIL-17C (R&D, Minneapolis, MN) or PBS every 3 days, respectively. For Treg differentiation experiments BALB/C recipients were injected with IL-17C-expresssing plasmid or vector control (60 ug/2 ml). 3 days later, recipients were lethally irradiated by X-Ray and transplanted with 1 × 10^∧^7 bone marrow (BM) cells and 3 × 10^∧^6 splenocytes from CD45.1 mice together with 5 × 10^∧^5 nTregs from FoxP3-eGFP mice or 1 × 10^∧^7 bone marrow (BM) cells from CD45.1 mice and 2 × 10^∧^6 naïve CD4^+^ T cells from FoxP3-eGFP mice. For haplo-identical aGVHD model, B6D2F1 mice received lethal irradiation by X-Ray at a dose of 950 cGy and were transplanted with 1 × 10^∧^7 bone marrow (BM) cells and 7.5 × 10^∧^7 splenocytes from C57BL/6 or IL-17C^−/−^ donors, respectively. Mice were monitored daily. Weight change and aGVHD symptoms were recorded every 3 days. Systemic aGVHD score was assessed by a cumulative scoring system as in previous reports (29). For histology examination, 2 weeks after transplantation, tissue was fixed in 10% formalin and embedded in paraffin for cutting into 5 μm sections and staining with hematoxylin and eosin (H&E). The histopathology score was assessed by a semi-quantitative scoring system as previously reported (16, 28).

### Plasmid construction

IL-17C was amplified from cDNA obtained from Hepa1-6 cell line and inserted into minicircle plasmid (pMC.EF1; SBI, Palo Alto, CA). Forward 5′-AGATCTATGAGTCTCCTGCTTCTAGGC-3′, reverse 5′-AGATCTTCACTGTGTAGACCTGGGAAG-3′. For *in vivo* overexpression, vector plasmid or minicircle-IL-17C plasmid was injected into BALB/C recipients by hydrodynamic gene transfer (HGT) 3 days before transplantation.

### Cell preparation and flow cytometry

Single-cell suspensions of the aGVHD target organs, including spleen, liver, lung, and intestine, were prepared as previously described (16). Antibodies used for flow cytometry staining including anti-CD69-PerCP/Cy5.5, anti-CD3-PE/CF594, anti-CD8-Pacific Blue, anti-CD4-APC/CY7, anti-CD25-PE, anti-FoxP3-APC, anti-CD4-PE/CF594, were purchased from BD Biosciences (Franklin lakes, NJ). Anti-IFN-γ-APC, anti-TNF-α-PE/CY7, anti-IL-17A-PerCP/Cy5.5, anti-H-2k^b^-PE, anti-H-2k^d^-FITC, anti-CD45.2-APC, anti-CD45.1-APC/CY7 were purchased from Biolegend (San Diego, CA). For hepatocytes isolation, single liver cells were resuspended in 50% Percoll solution, centrifuged at 2,000 rpm for 20 min. The purified anti-mouse CD16/32 antibody was purchased from eBioscience (San Diego, CA). Intercellular staining and Treg detection were performed by using CytoFix/CytoPerm buffer (BD Biosciences, San Diego, CA) and FoxP3 staining Kit (eBioscience, San Diego, CA), respectively according to the manufacturer's instructions. Samples were detected on a NovoCyte Flow Cytometer (ACEA Biosciences, San Diego, CA) and data were analyzed by using Flowjo software (Flowjo, Ashland, OR).

### Serum cytokine detection

Serum was harvested on day 14 post transplantation and stored at −80°C. Serum cytokine production, including IFN-γ, TNF, IL-2, IL-4, IL-6, IL-10, IL-17A, were measured by using a BD Th1/Th2/Th17 Cytometric Bead Array (CBA) kits on FACS CantoII Cytometry (BD Biosciences, San Diego, CA). Data were analyzed by BD FCAP Array software (BD Biosciences, San Diego, CA).

### Real-time PCR

Total RNA from aGVHD target tissues, including spleen, liver, lung and intestine, or sorted cell subsets was extracted using TRIzol reagent (Takara, Japan) and reverse transcribed into cDNA. Quantitative real-time PCR was performed using SYBR Green Master Mix (Applied Biosystems, Warrington, UK). All primers are listed as followers: β-actin, Forward 5′-ATCTGGCACCACACCTTC-3′, reverse 5′-AGCCAGGTCCAGACGCA-3′; Occludin, Forward 5′-AGACTACACGACAGGTGGGG-3′, reverse 5′-CTGCAGACCTGCATCAAAAT-3′; Zo-1, Forward 5′-GCAGACTTCTGGAGGTTTCG-3′, reverse 5′-CTTGCCAACTTTTCTCTGGC-3′; Claudin-1, Forward 5′-ACTGCCCTGCCCCAGTGGAA-3′, reverse 5′-TCAGCCCCAGCAGGATGCCA-3′; Claudin-4, Forward 5′-TCGCGCTTGGTAGCTGGTGC-3′, reverse 5′-GATCCCCAGCCAGCCCAGGA-3′; IL-6, Forward 5′-ACCAGAGGAAATTTTCAATAGGC-3′, reverse 5′-TGATGCACTTGCAGAAAACA-3′; IFN-γ, Forward 5′-GATGCATTCATGAGTATTGCCAAGT-3′, reverse 5′-GTGGACCACTCGGATGAGCTC-3′; IL-1β, Forward 5′-ACCTGTCCTGTGTAATGAAAGACG-3′, reverse 5′-TGGGTATTGCTAGGGATCCA; TNF, Forward 5′-AGGGTCTGGGCCATAGAACT-3′, reverse 5′-CCACCACGCTCTTCTGTCTAC-3′; IL-17C, Forward 5′-GCTCCTCCACACCTGCTAAC-3′, reverse 5′-CTGTGGGTAGCGGTTCTCAT-3′; IL-17RE, Forward 5′-CAGTCCCAGTGACGCTAGAC-3′, reverse 5′-ACCCATTAGAGCGGTGAGAG-3′. Housekeeping gene β-actin was used as an internal control. Relative expression levels of interested genes were calculated according to a ΔCt method.

### Treg differentiation assay

Plates were coated with 2 μg/ml anti-CD3 and 0.4 μg/ml anti-CD28 (eBioscience, San Digeo, CA) overnight at 4°C. Splenocytes from B6 WT mice and IL-17C^−/−^ mice were seeded 2 × 10^∧^5 cells/200 μl each well in the presence of 50 UI/ml rhIL-2 and 2.5 ng/ml rmTGF-β (Pepro Tech, Rosemont, IL) in RPMI 1640 medium (Gibco, Grand Island, NY) with 10% fetal bovine serum (BBI Life Sciences, Shanghai, China) for 72 h. Additionally,IL-17C^−/−^ splenocytes were treated with 200 ng/ml rmIL-17C or culture medium, respectively (R&D, Minneapolis, MN). For allo-antigen stimulated Treg polarizing assay, splenocytes from BALB/C mice were irradiated with 10Gy using X-Ray, then co-cultured with B6 WT or IL-17C^−/−^ splenocytes, respectively at a ratio of 1:3 for 72 h. Tregs differentiation was detected by using FoxP3 staining Kit (eBioscience, San Diego, CA). For experiments of iTreg differentiation, 1 × 10^∧^5 CD4^+^GFP^−^CD62L^+^CD44^−^ naïve CD4^+^ T cells were seeded into pre-coated plate (2 μg/ml anti-CD3 and 0.4 μg/ml anti-CD28) with or without 200 ng/ml rmIL-17C in the presence of 50 UI/ml rhIL-2 and 2.5 ng/ml rmTGF-β for 5 days. The indicated cells were sorted from FoxP3-eGFP mice by BD Melody (BD Biosciences, San Diego, CA).

### Depletion and blocking antibodies

Treg depletion were performed by using anti-CD25 antibodies (BioXCell,West Lebanon, NH) as previously described (30). Donor B6 WT and IL-17C^−/−^ mice were injected *i.v*. with 500 ug anti-CD25 or rat IgM isotype control on day −10, −7, −4. Consistent to previous studies, the efficiency of Treg depletion is about 60% detected by FACS. To block IL-6 signaling, recipient mice were intravenously administrated with 500 ug anti-mouse IL-6R or rat IgG2b antibody as control (BioXCell, West Lebanon, NH) on day −1, 3, 7. To block IFN-γ, mice were injected 250 ug anti-IFN-γ or rat IgG1 antibody as control (BioXCell,West Lebanon, NH) on day −1 and 6.

### Measurement of intestinal epithelia permeability

Mice were fasted for 4 h prior to deliver FITC-dextran (MW 4 kDa, 600 mg/Kg) via gavage 7 days post transplantation (Sigma, St. Louis, MO). Serums were harvested 4 h after dextran infusion. The FITC-dextran concentration was read at 480 and 520 nm with a SYNERGY HTX multi-mode reader (Bio-Tek, Winooski, VT). Permeability was normalized to an age-matched healthy mouse.

### Patients and sample collection

The study includes 68 patients with hematologic malignancies who underwent allo-HSCT at the First Affiliated Hospital of Soochow University in 2012. All patients were conditioned with the same myeloablative conditioning regimen, including high-dose cyclophosphamide (CTX) with busulfan. Serums were collected at the time of pre-conditioning. All patients have full clinical follow-up data. This study was approved by the Ethics Committee of Soochow University. Written informed consent was obtained from each patient in accordance with the Declaration of Helsinki.

### Statistical analysis

Murine aGVHD-related survival curves were plotted and analyzed by log-rank test using GraphPad Prism version 7 (GraphPad, San Diego, CA). Data were shown as mean±SEM. Paired Student *t*-test and one-way ANOVA with Dunnet's test were used for statistics of two groups or multiple comparisons, respectively. In human samples, receiver operator characteristic (ROC) curves were constructed for IL-17C level predicting the occurrence of aGVHD. The cutoff value was evaluated for sensitivity and specificity. The cumulative incidence of aGVHD was calculated by Gray's test. Factors that showed statistical significance (*P* < 0.05) in the univariate analysis were included in the multivariate analysis using Fine and Gray proportional hazards model. Overall survival (OS) was estimated using the Kaplan-Meier method, and compared between the groups using log-rank test. *P* value < 0.05 is considered significantly different (^*^P < 0.05; ^**^P < 0.01; ^***^P < 0.001).

## Results

### IL-17C mitigates murine aGVHD after allo-HSCT

To assess the possible role of IL-17C in the pathogenesis of aGVHD, we first examined the expressions of IL-17C and IL-17RE on day 3, 7, 12 post syngeneic and allogeneic HSCT by real-time PCR (Supplemental Figure 1). IL-17C expression was significantly increased in spleen, liver, lung, and intestine after both syngeneic and allogeneic HSCT (Supplemental Figure 1A). However, the IL-17C expression was lower in allogeneic than syngeneic HSCT hosts, suggesting the allogeneic immune response may inhibit the upregulation of IL-17C. IL-17RE expression was also up-regulated in the spleen, liver, and lung, but not intestine after HSCT, while stayed low after allo-HSCT.

**Figure 1:**
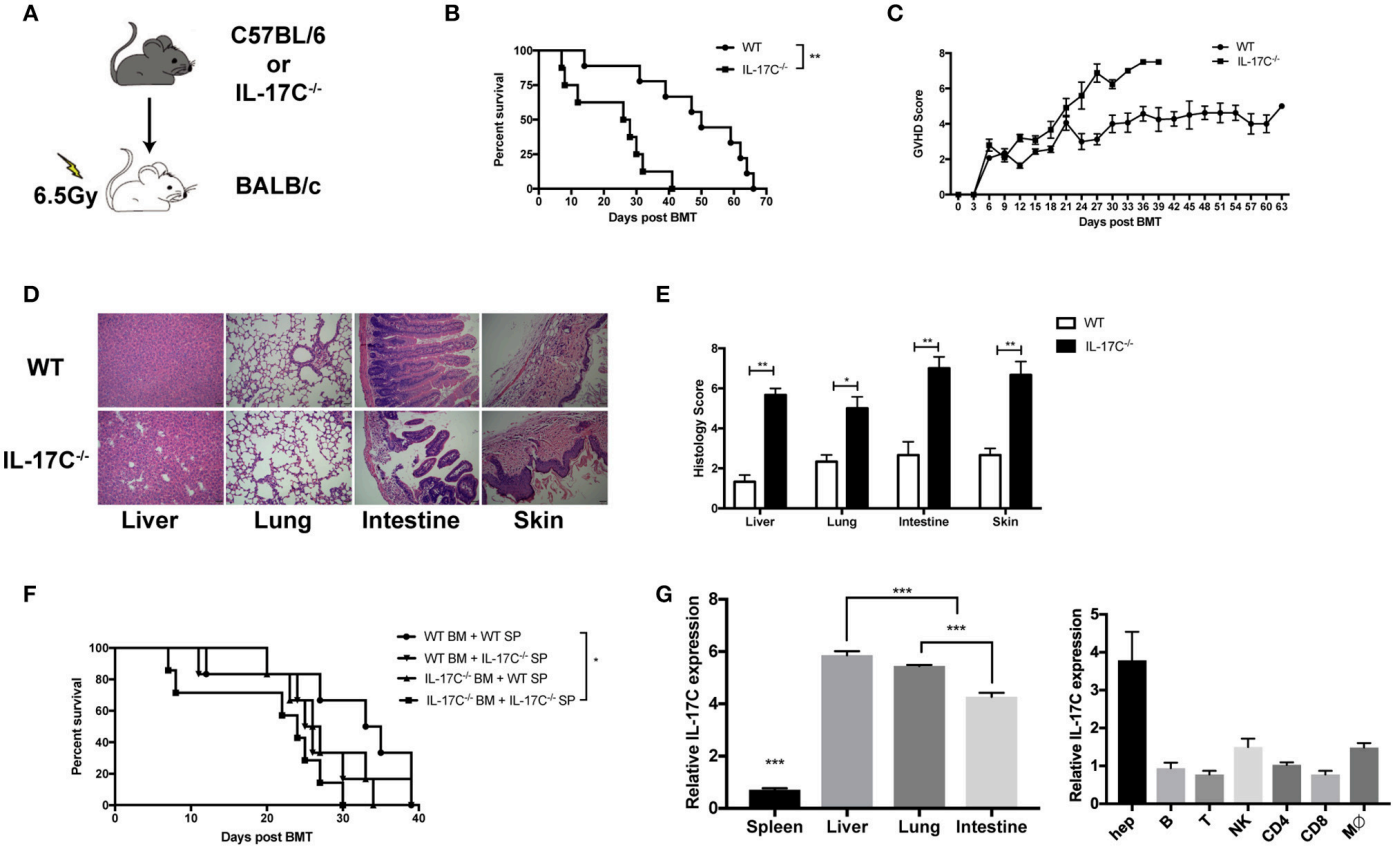
IL-17C mitigates murine aGVHD after allo-HSCT. BALB/c recipients were lethally irradiated and transplanted with 1 × 10^∧^7 bone marrow cells and 5 × 10^∧^6 splenocytes from B6 WT or B6 IL-17C^−/−^ mice respectively (**A–E**). (**A**) Schematic diagram of the experimental design; (**B**) aGVHD-related survival; (**C**) aGVHD clinical scores; (**D**) H&E staining; and (**E**) Histological analysis 14 days post-transplantation; (**F**) BALB/C recipients were transplanted with either B6 WT or IL-17C^−/−^ allografts of splenocytes and bone marrow cells as indicated; *n* = 6–7 per group. Survival was monitored and compared by log-rank test; (**G**) RNA from aGVHD target tissues (left) or the indicated cell subsets (right) were extracted and reversed into cDNA 2 weeks post transplantation. IL-17C expression was detected by real-time PCR. Data are representative of at least three experiments and presented as mean ± SEM. ^*^P < 0.05, ^**^P < 0.01, ^***^P < 0.001.

Next, we performed an MHC fully mismatched aGVHD model (B6 to BALB/C) to explore whether IL-17C could affect the development of aGVHD. BALB/C recipients were lethally irradiated with 650cGy by Co60 and transplanted with 1 × 10^∧^7 BM cells alone or with 5 × 10^∧^6 splenocytes from B6 WT mice or IL-17C^−/−^ mice, respectively (Figure 1A). Recipients of WT or IL-17C^−/−^ BM alone exhibited no aGVHD symptoms or aGVHD-related death (data not shown). Recipients of WT BMs and splenocytes survived for more than 60 days, while the recipients of IL-17C^−/−^ grafts all died within 40 days (*P* = 0.0016; Figure 1B). Recipients of IL-17^−/−^ grafts showed significantly higher aGVHD scores compared with WT controls (Figure 1C). Histologic assessment revealed more severe tissue damage in the liver, lung, small intestine, as well as skin in recipients with IL-17C^−/−^ grafts (Figures 1D,E). Moreover, in order to determine whether IL-17C produced by BM cells or splenocytes is required for aGVHD protection, we performed the transplant with either IL-17C^−/−^ BM or splenocytes together with the WT controls (Figure 1F). Recipients of either IL-17C^−/−^ BM/WT splenocytes or IL-17C^−/−^ splenocytes/WT BM developed a more severe aGVHD than WT controls, but had prolonged survival compared with the recipients of IL-17C^−/−^ BM/IL-17C^−/−^ splenocytes. In order to determine the cellular source of IL-17C, we then analyzed IL-17C expressions in recipient organs, as well as the donor-derived cell subsets (Figure 1G and Supplemental Figure 2). In recipients, IL-17C expression was significantly elevated in the liver (Figure 1G, left panel), especially in hepatocytes (Figure 1G, right panel). Donor-derived immune cell subsets exhibited relatively lower levels of IL-17C expression in both liver (Figure 1G right panel) and spleen (Supplemental Figure 2). Thus, our results demonstrated that IL-17C mitigated murine aGVHD and IL-17C derived from both donor BM and splenocytes, although produced at a relatively lower level compared with the host cells, contributed to its protective effect against aGVHD.

### IL-17C inhibits donor T cell responses during allo-HSCT

**Figure 2:**
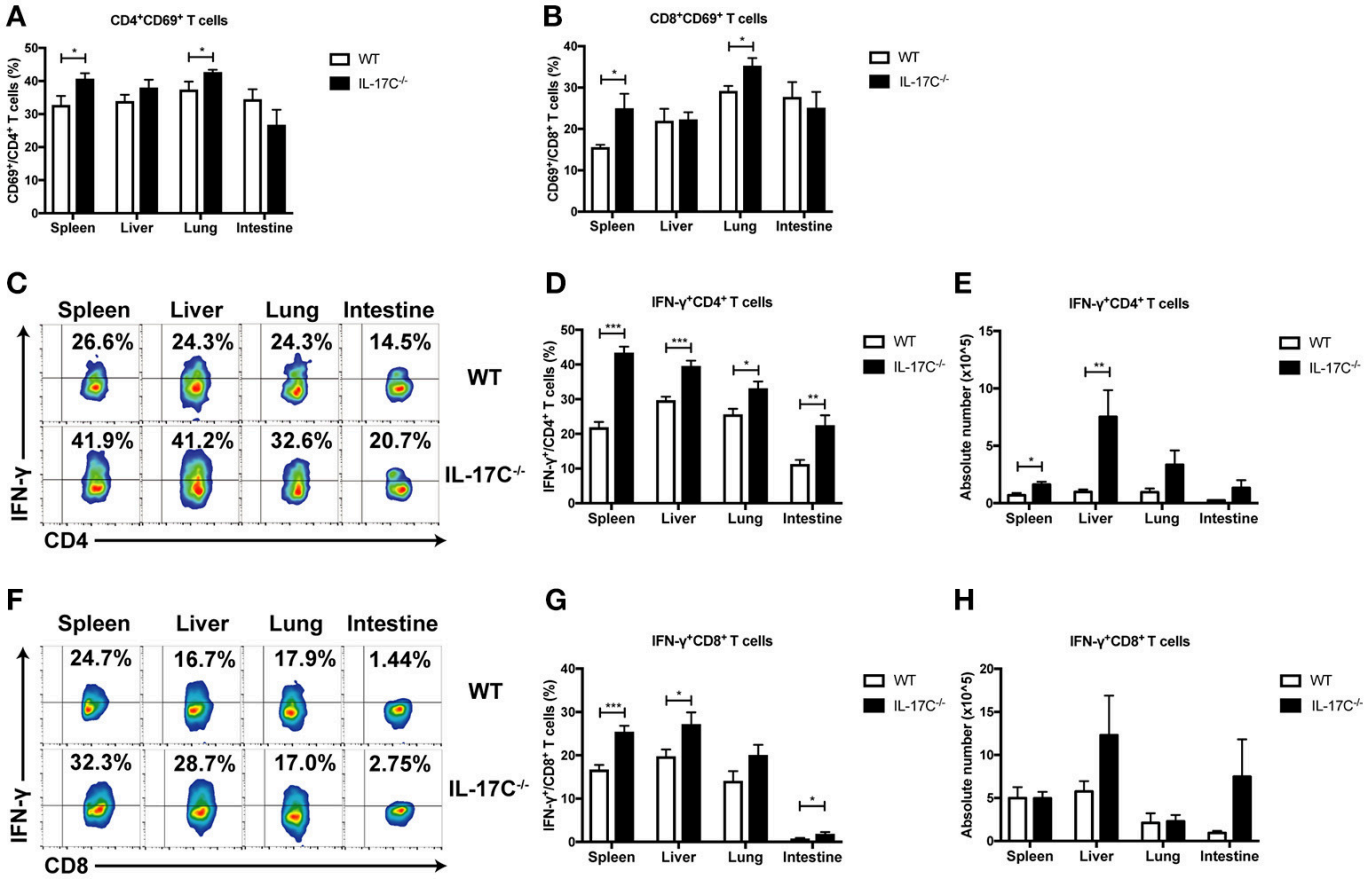
IL-17C inhibits donor T cell responses during allo-HSCT. BALB/c recipients were lethally irradiated and transplanted with 1 × 10^∧^7 bone marrow cells and 5 × 10^∧^6 splenocytes from WT or IL-17C^−/−^ mice, respectively. Percentages of donor T cell subsets were analyzed by FACS; *n* = 6–7 per group. Populations of activated CD4^+^ (**A**) and CD8^+^ (**B**) T cells were detected in spleen, liver, lung, and intestine 14 days post-transplantation. Populations of IFN-γ-producing CD4^+^ T (**C,D**) and CD8^+^ T cells (**F,G**) were examined by intracellular staining. Absolute cell numbers of Th1 and Tc1 cells were calculated (**E,H**). Data are representative of at least three experiments and presented as mean ± SEM. ^*^P < 0.05, ^**^P < 0.01, ^***^P < 0.001.

Allo-reactive donor T cell responses are the driving force for aGVHD. To investigate whether IL-17C can regulate T cell response, we analyzed T cell activation and cytokine production 14 days post-transplantation by flow cytometry. Percentages of CD69^+^CD4^+^ T cells and CD69^+^CD8^+^ T cells were significantly increased in spleen and lung in recipients of IL-17C^−/−^ graft (Figures 2A,B). Percentages of IFN-γ-producing CD4^+^ T cells in spleen, liver, lung, and intestine were also increased in the absence of IL-17C (Figures 2C,D). The numbers of CD4^+^IFN-γ^+^ T cells were significantly upregulated in spleen and lung in recipients of IL-17C^−/−^ graft (Figure 2E). Moreover, we observed an increase of CD8^+^IFN-γ^+^ T cell subsets in spleen, liver and intestine in recipients of IL-17C^−/−^ graft (Figures 2F,G). The numbers of CD8^+^IFN-γ^+^ T cells also showed a trend of increase in liver and intestine in recipients of IL-17C^−/−^ graft (Figure 2H). In addition, we examined the levels of inflammatory cytokines in serum (Supplemental Figure 3). In line with previous findings, we observed an increase of serum IFN-γ levels in recipients of IL-17C^−/−^ BM and splenocytes. The production of IL-6 and IL-17A were also significantly enhanced in the same group. Collectively, the results demonstrated that IL-17C could inhibit T cell activation and cytokine production in allo-HSCT recipients.

### Overexpression of IL-17C ameliorates aGVHD

**Figure 3:**
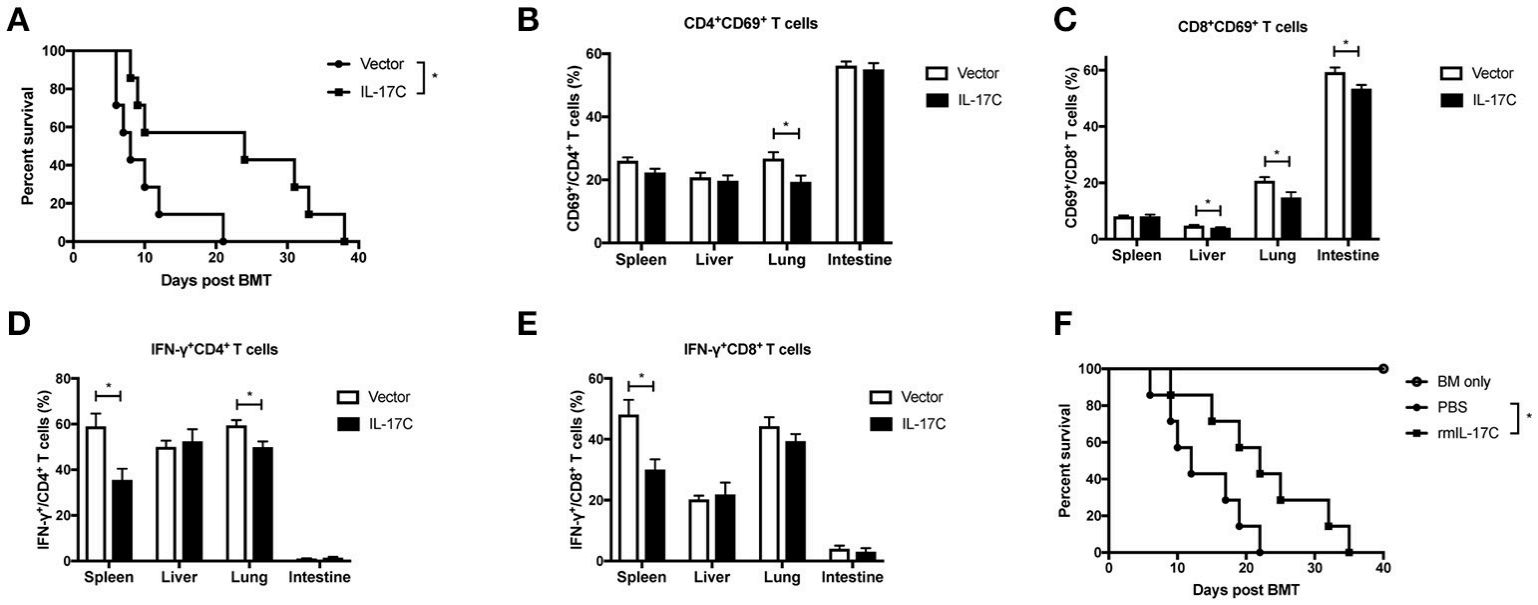
Overexpression IL-17C ameliorates aGVHD. Vector plasmid or minicircle-IL-17C plasmid was injected into BALB/C recipients by hydrodynamic gene transfer (HGT) 3 days before transplantation. Recipients were lethally irradiated and transplanted with 1 × 10^∧^7 bone marrow cells and 5 × 10^∧^6 splenocytes from IL-17C^−/−^ mice. (**A**) aGVHD-related survival; *n* = 7 per group. (**B**) Percentages of CD69^+^CD4^+^ T cells (**B**) and CD69^+^CD8^+^ T cells (**C**) in spleen, liver, lung, and intestine were detected on day 14 post-transplantation; *n* = 6 per group. Percentages of IFN-γ-producing CD4^+^ T (**D**) and CD8^+^ T (**E**) cells were examined by intracellular staining. (**F**) BALB/C recipients were transplanted with 1 × 10^∧^7 bone marrow cells together with 5 × 10^∧^6 splenocytes or 1 × 10^∧^7 bone marrow cells from IL-17C^−/−^ mice. Recipients were administrated with PBS or rmIL-17C every 3 days. BM only, *n* = 3; PBS, *n* = 7; rmIL-17C, *n* = 7. Data are representative of at least three experiments and presented as mean ± SEM. ^*^P < 0.05.

To further confirm the function of IL-17C in aGVHD development, recipient BALB/C mice were injected with IL-17C-expressing plasmid or control vector plasmid, respectively by hydrodynamic gene transfer (HGT) 3 days before transplantation. The expression of IL-17C was confirmed *in vivo* by real time PCR (Supplemental Figure 4). The mice were transplanted with 1 × 10^∧^7 IL-17C^−/−^ BMs and 5 × 10^∧^6 IL-17C^−/−^ splenocytes. As shown in Figure 3A, control mice developed severe aGVHD and died within 22 days. In contrast, about 40% recipient mice, which received IL-17C-expressing plasmid, survived more than 30 days. Next, we assessed T cell activation status and found that the activation of CD4^+^ T cells was significantly reduced in lung with IL-17C expression (Figure 3B). Moreover, we observed a significant reduction of CD8^+^ T cell activation in liver, lung and intestine (Figure 3C). Similarly, the percentages of Th1 cells were significantly decreased in spleen and lung (Figure 3D) and the Tc1 subsets were also decreased in spleen (Figure 3E) with IL-17C expression. Recombinant IL-17C injection further confirmed that IL-17C could prolong the aGVHD-related survival (Figure 3F). These data confirmed that IL-17C mitigates aGVHD and inhibits T cell activation and IFN-γ production after allo-HSCT.

### IL-17C maintains intestinal epithelia integrity and suppresses inflammation

**Figure 4:**
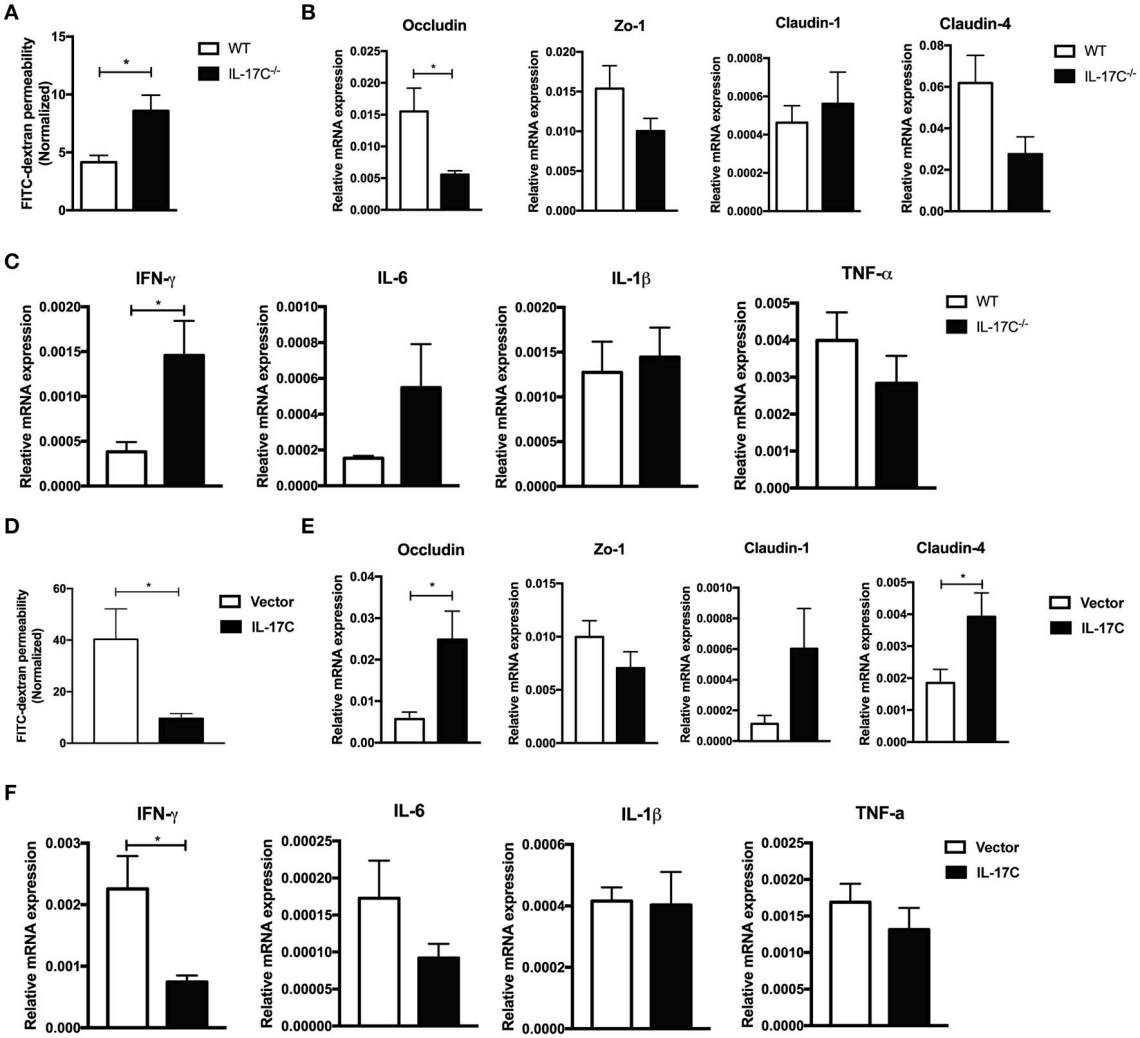
IL-17C maintains intestinal epithelia integrity and suppresses inflammation. BALB/c recipients were lethally irradiated and transplanted with 1 × 10^∧^7 bone marrow cells and 5 × 10^∧^6 splenocytes from WT or IL-17C^−/−^ mice, respectively. Recipients were fasted for 4 h prior to delivering 600 mg/Kg FITC-dextran via gavage 7 days post-transplantation. (**A**) The FITC-dextran concentration in serum was measured 4 h after infusion 7 days post-transplantation; *n* = 5 per group. (**B**) Total RNA from aGVHD target tissues was extracted and reversed into cDNA. Tight junction protein (**B**) and inflammatory cytokine (**C**) expressions were detected by real-time PCR; *n* = 4–5 per group. (**D**) BALB/c recipients were injected with vector plasmid or minicircle-IL-17C plasmid 3 days before transplantation, and then transplanted with 1 × 10^∧^7 bone marrow cells and 5 × 10^∧^6 splenocytes from B6 WT donors. Serum FITC-dextran concentration was measured on day 7 using the same method described before; *n* = 5 per group. Tight junction protein (**E**) and inflammatory cytokine (**F**) expressions in spleen, liver, lung, and intestine were detected by real-time PCR on day 7; *n* = 4 per group. Data are representative of at least three experiments and presented as mean ± SEM. ^*^P < 0.05.

Previous studies suggested the role of IL-17C in maintaining intestinal barrier functions (31), we then next investigated whether IL-17C could regulate intestinal epithelia integrity during allo-HSCT (Figure 4). We examined the intestinal permeability in recipients of WT or IL-17C^−/−^ graft 7 days post allo-HSCT. Recipients with IL-17C^−/−^ grafts showed significantly higher amount of FITC-dextran in the serum, indicating increased intestinal permeability (Figure 4A). Expression of tight junction protein occludin was significantly decreased in recipients with IL-17C^−/−^ grafts (Figure 4B). Zo-1 and Claudin-4 expressions were also showed slight decrease with no change in claudin-1 expression. IFN-γ expression was significantly increased in the intestine in recipients of IL-17C^−/−^ graft (Figure 4C). IL-6 expression was slightly elevated with no statistical difference. Thus, IL-17C may reduce aGVHD-related inflammation by maintaining intestinal barrier function.

To further confirm therole of IL-17C on the regulation of intestinal permeability and inflammation, we overexpressed IL-17Cin the host of allo-HSCT. IL-17C overexpression significantly reduced intestinal barrier permeability(Figure 4D). Tight junction proteins, including occludin and claudin-4, were markedly upregulated with IL-17C overexpression (Figure 4E). Moreover, we observed a substantial reduction of INF-γ and IL-6 expression (Figure 4F). These results demonstrated that IL-17C could maintain intestinal epithelia integrity and suppress inflammation.

### IL-17C promotes treg differentiation

**Figure 5:**
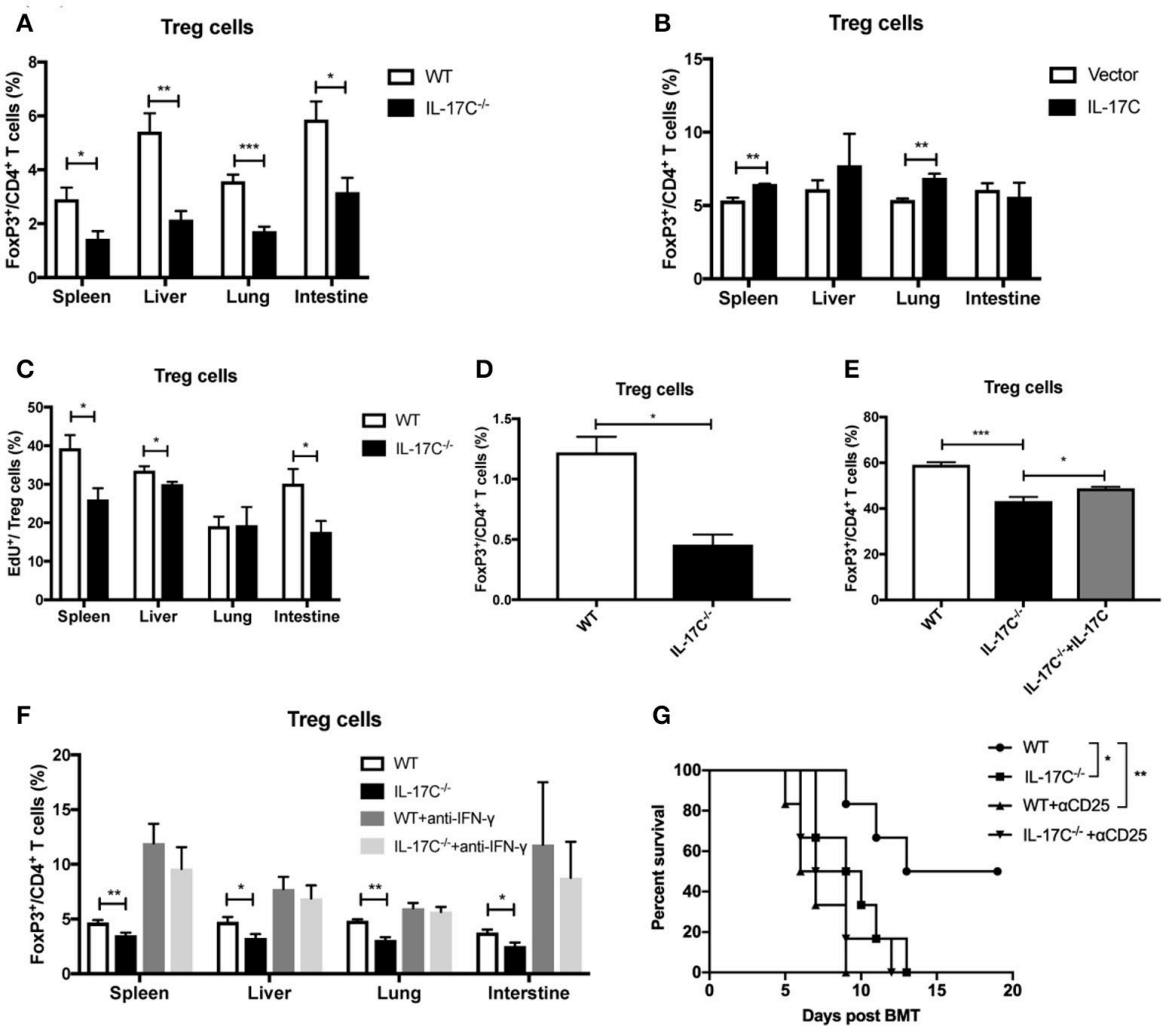
IL-17C promotes Treg cells differentiation. Lethally irradiated BALB/c recipients were transplanted with 1 × 10^∧^7 bone marrow cells and 5 × 10^∧^6 splenocytes from B6 WT and IL-17C^−/−^ donors, respectively. (**A**) Percentages of Treg cells in spleen, liver, lung, and intestine were detected on day 14 post-transplantation; *n* = 5–6 per group. (**B**) Percentages of Treg cells in BALB/c recipients IL-17C overexpression and vector control were detected on day 14 post-transplantation; *n* = 5–6 per group. (**C**) BALB/c recipients were transplanted with B6 WT and IL-17C^−/−^ allografts respectively, and injected with 5 mg/kg EdU 4 h prior to detection on day 14 post-transplantation. EdU incorporation was analyzed by flow cytometry; *n* = 6 per group. (**D**) Irradiated BALB/C splenocytes were co-cultured with B6 WT and IL-17C^−/−^ splenocytes, respectively at a ratio of 1:3 for 72 h. Differentiation of Treg cells was examined by flow cytometry. (**E**) Plate was pretreated with anti-CD3 and anti-CD28 overnight at 4°C. Splenocytes from B6 WT mice and IL-17C^−/−^ mice were seeded into 96-well plate in the presence with rhIL-2 and rmTGF-β. Percentage of Treg cells was examined 72 h later by flow cytometry. (**F**) Recipients were administered with anti-IFN-γ or rat IgG1 antibody on day−1 and 6. Recipients were transplanted with WT or IL-17C^−/−^ allografts on day 0; *n* = 5–6 per group. Percentages of Treg cells in spleen, liver, lung, and intestine were detected on day 12–14. (**G**) B6 WT and IL-17C^−/−^ donors were injected with anti-CD25 or rat IgM isotype control on day −10, −7, −4. BALB/c recipients were lethally irradiated and received B6 WT, IL-17C^−/−^, B6 Treg depleted, and IL-17C^−/−^ Treg depleted grafts. aGVHD-related survival was monitored; *n* = 6 per group. Data are representative of at least three experiments and presented as mean ± SEM. ^*^P < 0.05, ^**^P < 0.01, ^***^P < 0.001.

Treg cells play a vital role in maintaining immune-tolerance, preventing autoimmune diseases and limiting inflammatory diseases, including aGVHD (30, 32–34). In autoimmune hepatitis, IL-17C^−/−^ mice displayed a significant reduction of CD25 expressions on T cells (26). Therefore, IL-17C may inhibit aGVHD pathogenesis through regulation of Tregs. Indeed, in our aGVHD model, there was a reduction in Treg percentages in spleen, liver, lung and intestines of recipients of IL-17C^−/−^ graft compared with those of WT graft (Figure 5A), indicating IL-17C may promote Treg differentiation or maintain its stability *in vivo*. When we overexpressed IL-17C in the host of allo-HSCT, percentages of Treg cells were significantly elevated in spleen and lung when compared to vector controls (Figure 5B). In order to determine whether the reduced Tregs populations in recipients of IL-17C^−/−^ graft were due to the decreased proliferation, we examined Treg cell proliferation *in vivo* (Figure 5C). As expected, we found significantly reduced EdU^+^ Treg cells in recipients of IL-17C^−/−^ graft in spleen, liver and intestine. Then we performed mixed lymphocyte reactions with B6 WT or IL-17C^−/−^ splenocytes as the responders and BALB/C splenocytes as the stimulators and determined the percentages of Treg cells in the reaction. The percentage of Treg cells was significantly reduced in IL-17C^−/−^ responders (Figure 5D). *In vitro* Treg differentiation assay also revealed that IL-17C deficiency significantly inhibited Treg differentiation, whilst exogenous IL-17C treatment could restore the Treg percentages (Figure 5E). IFN-γ has been reported to inhibit Treg functions and differentiations (35–37). The reduced Treg percentages in recipients of IL-17C^−/−^ graft was restored to the same levels as those of WT recipients when IFN-γ was neutralized (Figure 5F), indicating an important role of IFN-γ in IL-17C-regulated Treg differentiation.

To further investigate whether IL-17C could inhibit aGVHD through promoting Treg differentiation and expansion, we depleted donor-derived Treg cells by using anti-CD25 antibody as described previously (30). Consistent with previous findings (30), depletion of donor-derived Treg cells significantly accelerated the aGVHD-associated mortality (Figure 5G). IL-17C deficiency did not further aggravate aGVHD in the absence of Treg cells. Together, these results indicated that IL-17C promoted Treg differentiation and expansion in an IFN-γ dependent manner, which was essential in mitigating murine aGVHD.

### IL-17C regulates both iTreg and nTreg subsets during aGVHD

**Figure 6:**
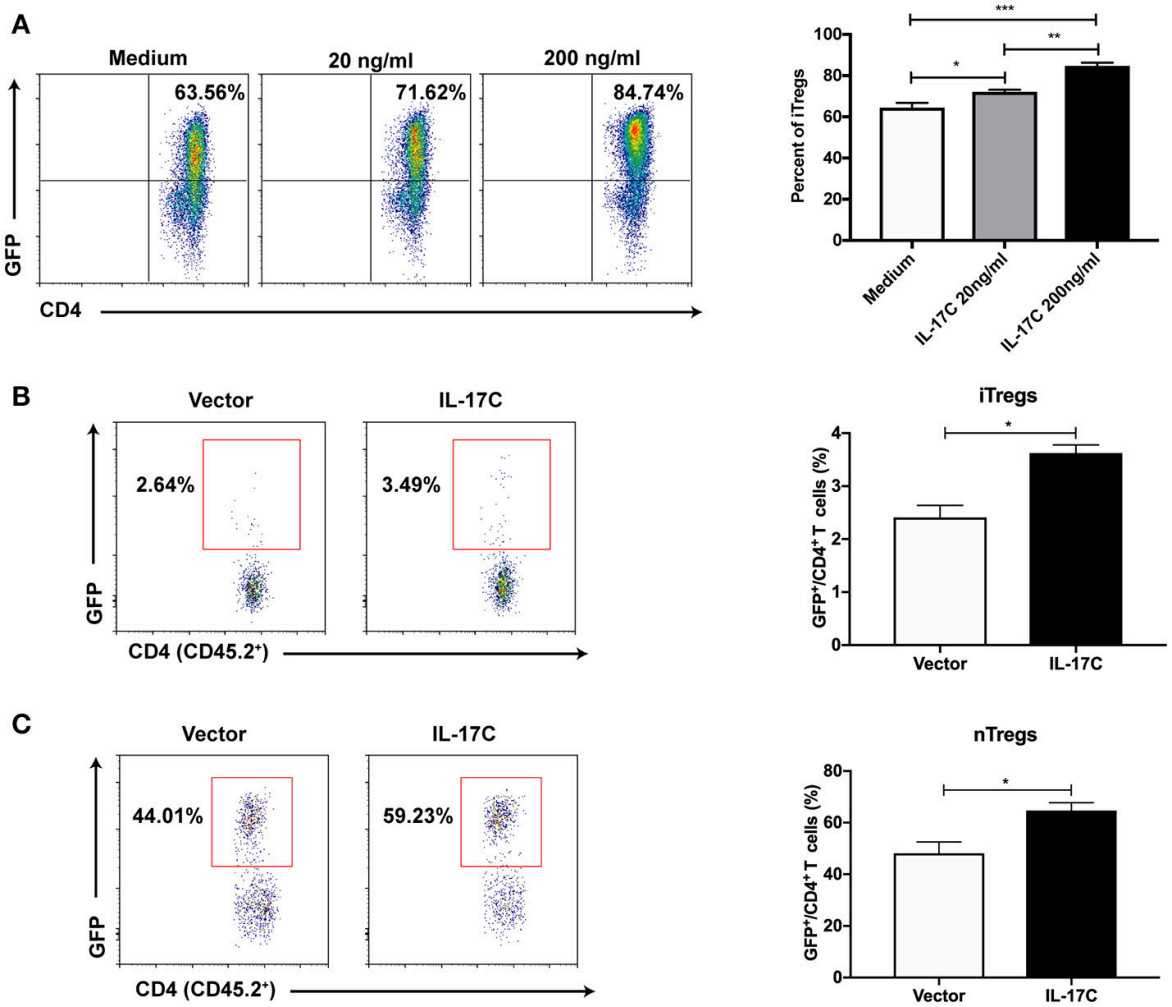
IL-17C regulates both iTreg and nTreg cells during aGVHD. (**A**) Plate was coated with anti-CD3 and anti-CD28 overnight at 4°C. Naïve CD4^+^ T cells (CD4^+^CD62L^+^CD44^−^GFP^−^) were sorted from FoxP3-eGFP mice and seeded into 96-well plate in the presence or absence with rmIL-17C. Generation of iTreg cells were induced by rhIL-2 combined with rmTGF-β and examined 5 days later by flow cytometry. (**B**) BALB/C recipients were injected with IL-17C or control plasmid. 3 days later, recipients were lethally irradiated and transplanted with 1 × 10^∧^7 bone marrow cells from CD45.1 mice and 2 × 10^∧^6 naïve CD4^+^ T cells (CD45.2^+^CD4^+^CD62L^+^CD44^−^GFP^−^) from FoxP3-eGFP mice. Generation of iTregs were determined in H2-Kb^+^CD45.2^+^CD45.1^−^CD4^+^ populations at 10 days post transplantation in spleen; *n* = 5 per group. (**C**) BALB/C recipients were hydrodynamically injected with IL-17C plasmid or vector control. 3 days later, recipients were lethally irradiated and transplanted with 1 × 10^∧^7 bone marrow cells together with 3 × 10^∧^6 splenocytes from CD45.1 mice and 5 × 10^∧^5 nTreg cells (CD45.2^+^CD4^+^GFP^+^) from FoxP3-eGFP mice. GFP expression was determined in H2-Kb^+^CD45.2^+^CD45.1^−^CD4^+^ populations at 10 days post transplantation in spleen; *n* = 3 per group. Data are representative of two experiments and presented as mean ± SEM. ^*^P < 0.05, ^**^P < 0.01, ^***^P < 0.001.

To further investigate the role of IL-17C on Treg cells, we induced iTreg cells from FoxP3-GFP^−^ naïve CD4^+^ T cells from FoxP3-eGFP reporter mice *in vitro* in the presence or absence of IL-17C. IL-17C significantly promoted the generation of iTreg cells in a dose-dependent manner (Figure 6A). To evaluate the role of IL-17C on iTreg cells *in vivo* in the setting of aGVHD, we first overexpressed IL-17C with minicircle plasmid and adoptively transferred CD45.1^+^ BMs together with CD45.2^+^ FoxP3-GFP^−^ naïve CD4^+^ T cells. We found that iTreg percentages were significantly increased in IL-17C overexpressing recipients (Figure 6B). With respect to the nTregs, recipients with IL-17C expression received CD45.1^+^ BMs and splenocytes together with CD45.2^+^ FoxP3-GFP^+^CD4^+^ nTreg cells. A substantial population of nTregs lost FoxP3 expression during aGVHD, whereas IL-17C significantly sustained FoxP3 expression (Figure 6C). Therefore, IL-17C promotes both iTreg and nTreg subsets in aGVHD.

### IL-17C expression level predicts aGVHD incidence and severity in allo-HSCT patients

**Figure 7:**
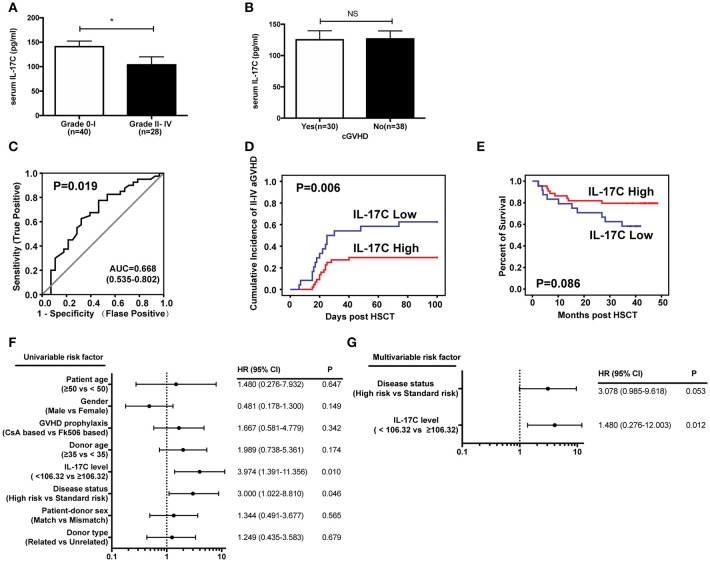
IL-17C expression level predicts aGVHD incidence and severity in allo-HSCT patients. Expressions of IL-17C in human allo-HSCT patients during pre-conditioning were measured by ELISA. (**A**) IL-17C expression in aGVHD patients. (**B**) IL-17C expression in cGVHD patients. (**C**) Sensitivity and specificity of the analysis. The area under the ROC curve (AUC) was 0.668. The cutoff value used from the ROC curve was 106.32 pg/ml. Sensitivity and specificity was 77.5 and 53.6%, respectively. (**D**) The cumulative incidence of grade II-IV aGVHD was significantly lower in patients with high IL-17C expression levels by using Gray's test. (**E**) IL-17C-high patients showed prolonged survival compared to those with low IL-17C expression with Kaplan-Meier survival by log-rank test. (**F**) Univariate analyses revealed that IL-17C levels < 106.32 pg/ml were significantly associated with grade II-IV aGVHD. (**G**) Fine and Gray proportional hazards model analysis confirmed that low IL-17C level was the strongest parameter associated with II-IV aGVHD. ^*^P < 0.05.

To determine the clinical relevance of IL-17C in aGVHD patients, we examined IL-17C production in serums from 68 patients during pre-conditioning. The donor and patient characteristics are showed in Table 1. Among them, 26 patients (38.2%) developed grade I aGVHD, 19 patients (27.9%) developed grade II aGVHD, 5 patients (7.4%) developed grade III aGVHD, and 4 patients (5.9%) developed grade IV aGVHD. Serum IL-17C levels were significantly decreased in grade II-IV aGVHD patients compared to grade 0-I aGVHD patients (Figure 7A). Total of 30 patients (44.1%) had cGVHD. However, IL-17C expression levels were comparable between cGVHD patients and non-cGVHD patients (Figure 7B). IL-17C serum level during pre-conditioning could predict grade II-IV aGVHD (AUC = 0.668, 95% CI = 0.535-0.802, *P* = 0.019) when we used 106.32 pg/ml as cutoff value from the ROC curve (Figure 7C). Next, patients were divided into two groups according to the cutoff value. Cumulative incidence of grade II-IV aGVHD was significantly lower in IL-17C high patients (IL-17C≥106.32 pg/ml) than in IL-17C low patients (IL-17C < 106.32 pg/ml) (Figure 7D). Patients with high IL-17C expression had prolonged survival than patients with low IL-17C expression (Figure 7E). To determine the predictive value of IL-17C in the occurrence of grade II-IV aGVHD, we performed univariate analysis and found that IL-17C expression level < 106.32 pg/ml (HR = 3.974, 95% CI = 1.391-11.356, *P* = 0.010) and high risk of disease status (HR = 3.000, 95% CI = 1.022-8.810, *P* = 0.046) were significantly associated with higher aGVHD incidences (Figure 7F). Multivariate analysis further confirmed that low IL-17C level (HR = 1.480, 95% CI = 0.276-12.003, *P* = 0.012) was the strongest parameter associated with grade II-IV aGVHD (Figure 7G). These results demonstrated that low IL-17C expression level could be an independent risk factor for predicting grade II-IV aGVHD.

**Table 1:** The association of IL-17C levels at pre-conditioning with clinical factors.

**Factors**	**Total**	**IL-17c ≥ 106.32**	**IL-17c < 106.32**	***P* value**
Age median	28 (3–59)	27.5 (3–59)	29 (13–52)	0.904
**GENDER**				
Male	41	25	16	0.428
Female	27	19	8
Donor age	36 (16–55)	37 (16–52)	35 (18–55)	0.887
**PATIENT-DONOR**				
Match	44	30	14	0.417
Mismatch	24	14	10
**GVHD**				
CsA based	48	32	16	0.189
Fk506 based	20	12	8
**DONOR TYPE**				
Related	48	32	16	0.600
Unrelated	20	12	8
**DIAGNOSIS**				
AML	29	20	9	0.263
ALL	23	14	9
MDS	6	2	4
CML	10	8	2
**DISEASE STATUS**				
Standard risk	48	32	16	0.600
High risk	20	12	8
**aGVHD GRADE**				
0	14	11	3	0.005
I	26	20	6
II	19	11	8
III	5	1	4
IV	4	1	3
**PROGNOSIS**				
Survival	49	35	14	0.173
Replase	8	4	4
Other	11	5	6

## Discussion

The protective role of IL-17A in aGVHD after allo-HSCT has been characterized (15–21), while the function of IL-17C in aGVHD remains unknown. In the current study we utilized the IL-17C-deficient graft and IL-17C overexpression model to investigate the effect of IL-17C on murine aGVHD and further analyzed its potential application in human aGVHD patients. To our knowledge, this is the first report to show IL-17C deficiency resulted in aggravated aGVHD, whilst IL-17C overexpression significantly prolonged aGVHD-associated survival. T cells showed enhanced activation and cytokine production in the hosts that received IL-17C-deficient grafts, while IL-17C overexpression reduced T cell activation and cytokine production. Moreover, IL-17C deficiency resulted in increased intestinal permeability and amplified inflammation, while IL-17C overexpression showed the opposite results. Further studies demonstrated IL-17C might inhibit aGVHD by promoting donor-derived Treg differentiation in an IFN-γ dependent manner. In human aGVHD patients, IL-17C expression is reduced in more severe aGVHD and low IL-17C expression level is significantly associated with grade II-IV aGVHD incidence and could be an independent risk factor for aGVHD.

IL-17C has been reported to be produced by intestinal epithelial cells after flagellin stimulation (23). Microbiota can also drive IL-17C expression in intestinal epithelial cells (38). Moreover, IL-17C is significantly up-regulated in hepatocytes treated by Con-A (26). In our murine aGVHD model, we investigated IL-17C expression after syngeneic and allogeneic HSCT and found although IL-17C expression was increased in both situations, its expression was significantly lower in allo-HSCT when compared to syngeneic HSCT. Next, we demonstrated that both splenocyte- and BM-derived IL-17C contributed to the protective effect of IL-17C. Although produced at a relatively lower level by the donor-derived immune cells compared with the host cells, donor-derived IL-17C is still important in mitigating aGVHD in our murine aGVHD model. The underlining mechanism needs further investigation. We also performed haplo-identical aGVHD model to explore the function of IL-17C. However, we didn't observe any difference in the survival between recipients of WT and IL-17C^−/−^ grafts (Supplemental Figure 5), which may be due to the reduced allo-reactivity of the donor T cells and retained Treg function in haplo-identical model. A recent study revealed that the suppressive function of iTreg in haplo-identical model was less marked than that in the MHC-fully mismatched aGVHD model (39). It has also been reported that most nTreg cells retained FoxP3 expression in syngeneic BMT, whereas they lost FoxP3 expression in allogenic transplantation (40, 41). As nTreg is more stable than iTreg in FoxP3 expression, *in vitro* experiments for inducing loss of FoxP3 expression for nTregs required a high concentration of inflammatory cytokines (41, 42). Therefore, the loss of suppressive function of both iTreg and nTreg may be less in the haplo-identical model than the MHC-fully mismatched aGVHD model, possibly due to reduced allo-reactivity of the donor T cells and inflammation. IL-17RE is identified as the unique functional receptor for IL-17C (22–24). The host IL-17RE expression was also increased in the spleen, liver and lung, but not intestine. The IL-17RE expression level became higher on day 12 after allo-HSCT than syngeneic HSCT, which could be a compensatory increase due to the lower IL-17C expression. The significance of IL-17RE expression in the aGVHD target organs also needs further investigation.

T cell activation in recipients of IL-17C-deficient grafts was elevated compared to that in WT recipients. However, CD8^+^ T cells activation was significantly reduced in IL-17C^−/−^ mice after ConA stimulation in autoimmune hepatitis (26). We also observed the IFN-γ production in CD4^+^ and CD8^+^ T cells were significantly elevated in recipients of IL-17C^−/−^ graft. No differences were detected in the TNF-α productions between recipients of WT and IL-17C^−/−^ graft. However, IL-17C was firstly reported for the induction of TNF-α production in THP-1 cells (25). Besides, IL-17C also promoted TNF-α production after *C. rodentium* and *C. albicans* infection (24, 27). Although we found increased IFN-γ mRNA level in the intestine and elevated IL-6 and IFN-γ serum levels in hosts receiving IL-17C^−/−^ graft, decreased IL-6 and IFN-γ productions were detected in hepatitis with IL-17C deficiency (26). IL-17C does not directly affect effector T cell function (data not shown). Its effect on T cell activation and cytokine production may heavily depend on the status of the myeloid cell populations in certain disease conditions. Therefore, the regulatory role of IL-17C on T cells and inflammatory cytokine productions may be largely dependent on the disease settings.

IL-17C has been reported to maintain intestinal stability through tight junction formation (31). Expression and localization of tight junction proteins occludin and Zo-1 were critical for aGVHD prevention (43). We also found altered expression of tight junction proteins and increased intestinal permeability after allo-HSCT in the absence of IL-17C. Thus, IL-17C may promote intestinal barrier function through upregulating the expression of tight junction proteins. However, further studies are needed to characterize the signaling pathways involved in this process.

Previous studies have demonstrated that Treg cells can effectively prevent aGVHD by secreting IL-10 and directly suppressing donor T cell expansion (44, 45). We found reduced percentages of Tregs in recipients of IL-17C^−/−^ grafts. As predicted, an increase of Treg cells was observed with IL-17C overexpression. Depletion of Treg cells in the graft diminished the protective effect of IL-17C in aGVHD model. Although we found IFN-γ production is essential for the decreased Treg percentages in recipients of IL-17C^−/−^ graft, other studies suggest Treg-derived IFN-γ is critical for its suppressive capacity (46, 47). Thus, Treg intrinsic IFN-γ may play an opposite role from that produced by conventional CD4^+^ T cells. IL-6 promotes the activation of signal transducer and activator of transcription (STAT)-3 (48), which is an important inhibitor of Treg differentiation in aGVHD (40, 41). The precise role of IL-6/IL-6R signaling on Treg differentiation in the course of aGVHD is controversial. One group demonstrated that blockade of IL-6 signaling augmented Treg reconstitution (49), while another group found that inhibition of IL-6 signaling did not alter the absolute number of Treg cells (50). We observed increased IL-6 production in the recipients of IL-17C^−/−^ grafts, which led us to assess the possibility of IL-17C affecting Treg differentiation through IL-6 in our aGVHD model. However, we found IL-6/IL-6R signaling is dispensable for the decreased Treg differentiation observed in recipients of IL-17C^−/−^ graft (Supplemental Figure 6). Increased IFN-γ production may also inhibit Treg differentiation, as IFN-γ has been reported to promote Treg fragility (37).

Serum IL-17C expression level significantly predicts aGVHD incidence and was decreased in grade II-IV aGVHD patients compared to grade 0-I aGVHD patients (Figure 7A). However, it was comparable between cGVHD patients and non-cGVHD patients (Figure 7B). This may be due to the differences in pathogenesis between aGVHD and cGVHD. aGVHD is mostly driven by allo-reactive T cells, but chronic GVHD is possibly mediated by both allo- and auto-reactive T cells (51). cGVHD has been recently recognized as a Th17/Tc17, Tfh, and TGF-β-producing macrophages-mediated disease (52, 53). The aberrant of germinal center B cell reaction and antibody formation are also critical for cGVHD (54). However, the effect of IL-17C on B cell functions remains unknown. Studies are needed to further uncover the function of IL-17C in cGVHD in preclinical models.

Taken together, our study provides the first evidence that IL-17C can regulate the immune responses and pathogenesis of aGVHD. It may mitigate aGVHD through promoting epithelial barrier function and Treg differentiation. In allo-HSCT patients, IL-17C may serve as a novel prognosis marker and potential therapeutic target for aGVHD.

## Author contributions

DW and HaL designed the study. HG, SM, and SL performed the research. YoL, ZJ, YZ, YS, LL, BH, YuL, YW, YX, and YM contributed to the experiments. HG analyzed the data. SL collected and analyzed the clinical data. HoL provided the blood samples and patient data. CD provided the IL-17C^−/−^ mice. HG, DW, and HaL wrote the manuscript.

### Conflict of interest statement

The authors declare that the research was conducted in the absence of any commercial or financial relationships that could be construed as a potential conflict of interest.
