# Supplementary material for: IL-17C Mitigates Murine Acute Graft-vs.-Host Disease by Promoting Intestinal Barrier Functions and Treg Differentiation
Source: Front Immunol. 2018 Nov 26;9:2724. doi: 10.3389/fimmu.2018.02724 (PMC6275224; doi:10.3389/fimmu.2018.02724)
Supplement: Supplementary file 1 [file Data_Sheet_1.docx]

***Supplementary Material***

**IL-17C mitigates murine acute graft-versus-host disease by promoting intestinal barrier functions and Treg differentiation**

Huanle Gong, *^,1^Shoubao Ma, *^,1^Shuangzhu Liu, *^,1^Yonghao Liu, * Ziqi Jin, * Ying Zhu, * Yuan Song, † Lei Lei, * Bo Hu, * Yu Mei, † Hong Liu, * Yuejun Liu, * Yan Wu, §Chen Dong, ‡ Yang Xu, * Depei Wu, * and Haiyan Liu^†^

Corresponding author:

Dr. Haiyan Liu, Email: [micliuh@nus.edu.sg](mailto:micliuh@nus.edu.sg)

Or

Dr. Depei Wu, Email: [wudepei@medmail.com.cn](mailto:wudepei@medmail.com.cn)

**
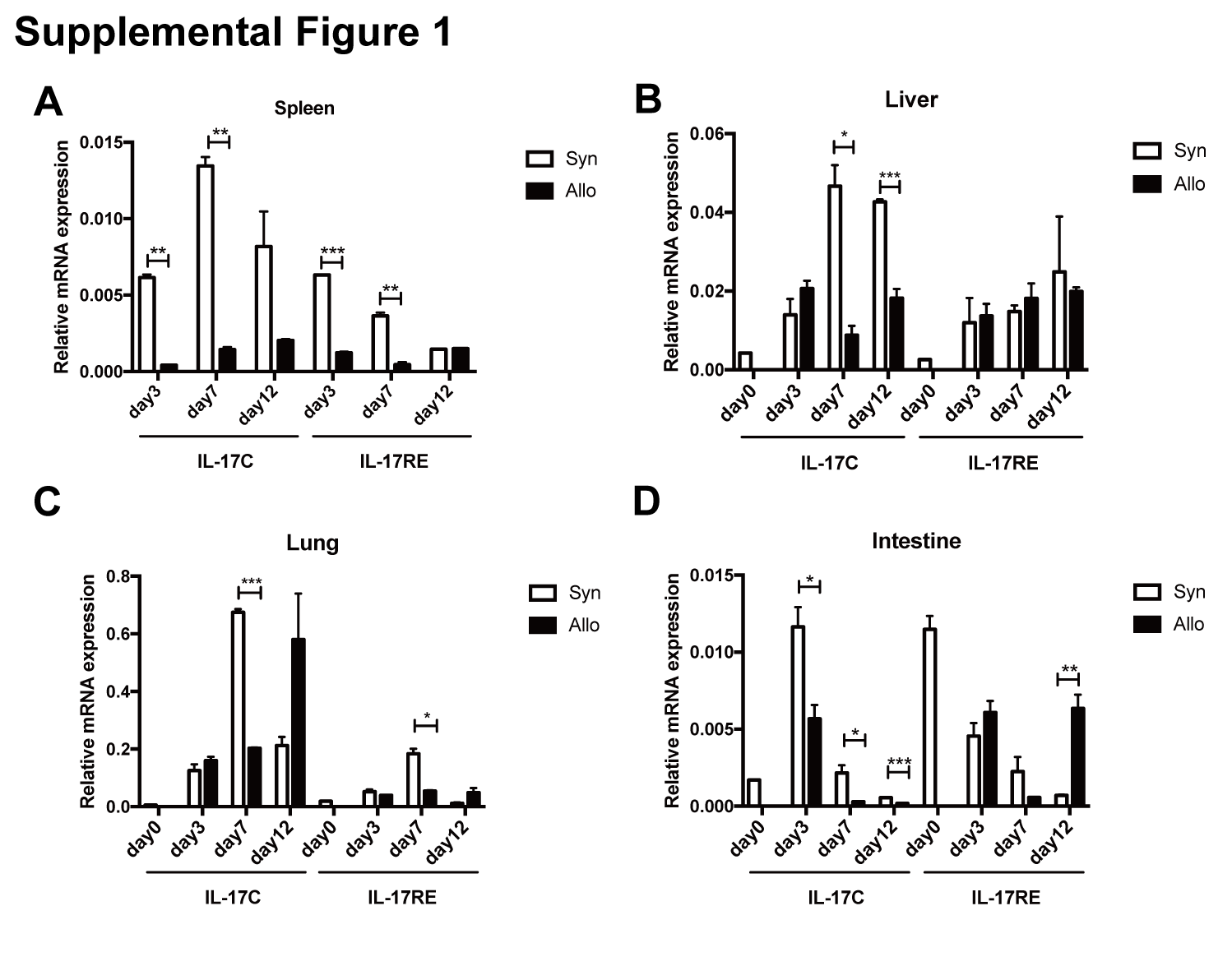
**

**Supplemental Figure 1.** IL-17C and IL-17RE expressions after syngeneic and allogeneic HSCT. Lethally irradiated BALB/c recipients were transplanted with 1x10^7 bone marrow cells and 5x10^6 splenocytes from C57BL/6 (allogeneic) or BALB/c (syngeneic) donors respectively (A-D). The relative mRNA expression levels of IL-17C and IL-17RE in spleen (A), liver (B), lung (C), and intestine (D) were detected by real-time PCR on day 3, 7, and 12 post transplantation; n=3-5 per group. Data are representative of at least three experiments and presented as mean ± SEM. **P*< 0.05, ***P*< 0.01, ****P*< 0.001.

**
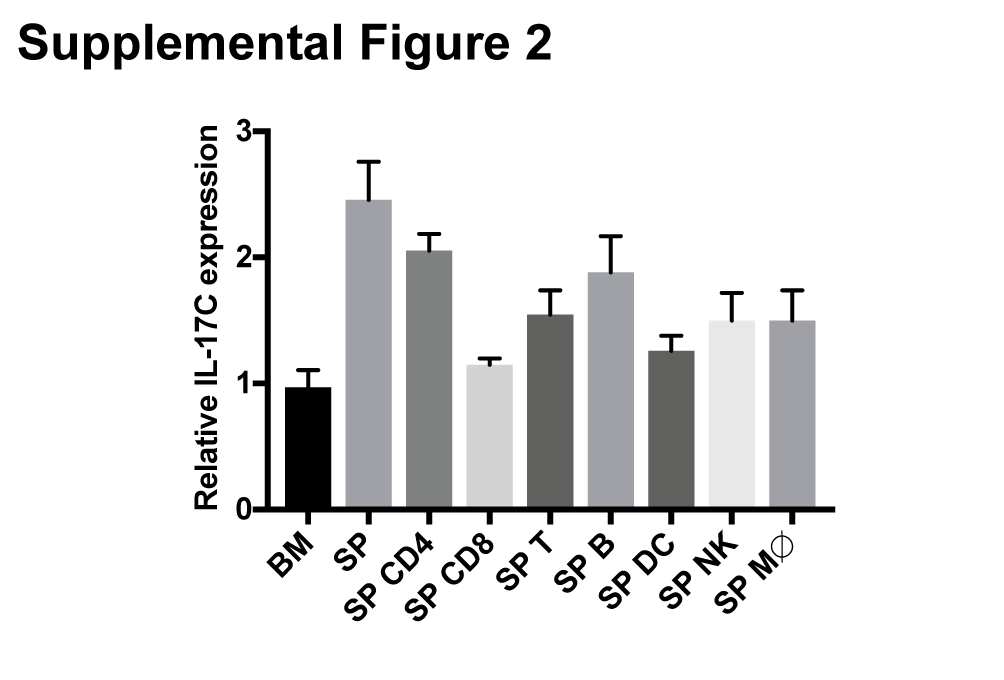
**

**Supplemental Figure 2.** RNA from the indicated cell types of C57BL/6 donors were extracted and reversed into cDNA. IL-17C expression was detected by real-time PCR. Data are representative of two experiments and presented as mean ± SEM.

**
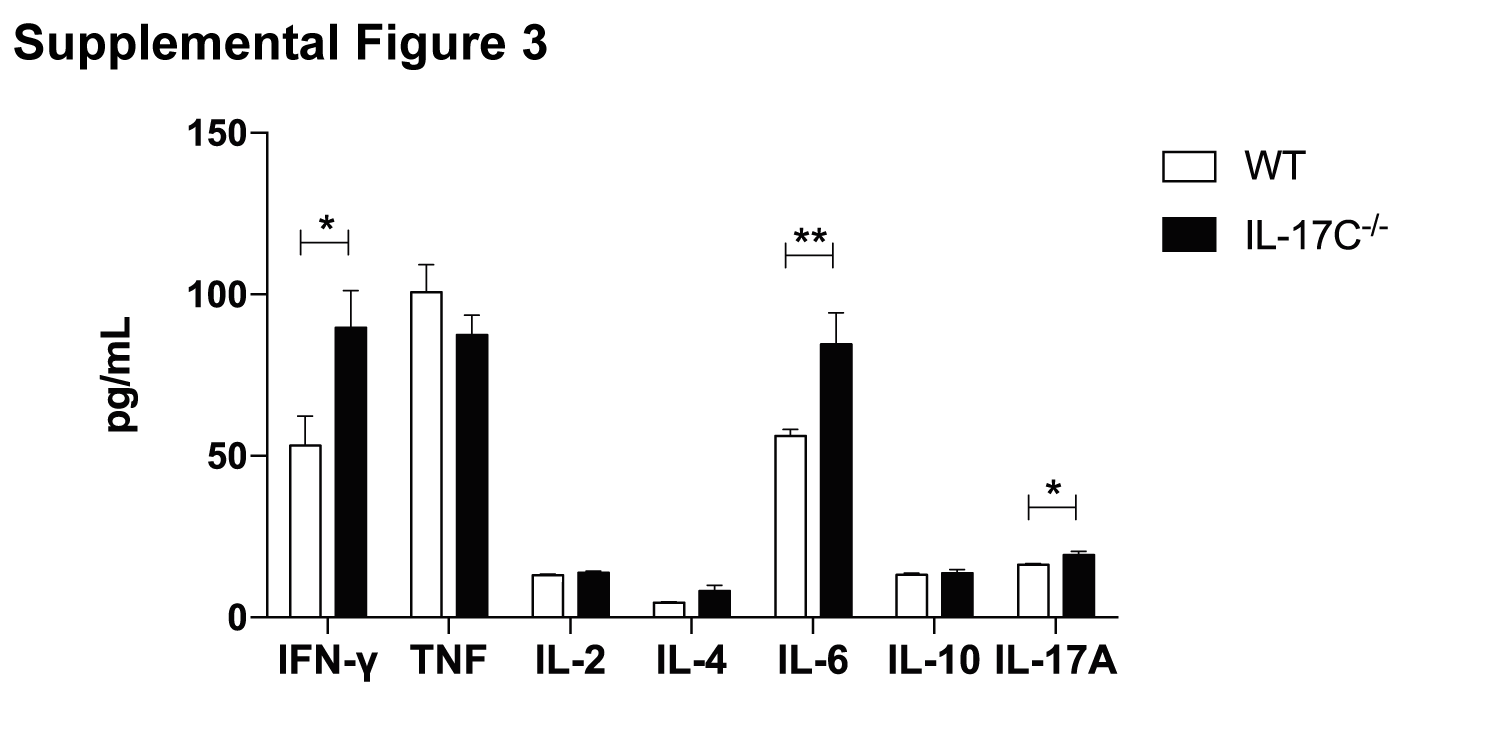
**

**Supplemental Figure 3.** Proinflammatory cytokine productionin the serum of WT and IL-17^-/-^ recipients. Lethally irradiated BALB/c recipients were transplanted with 1x10^7 BM cells and 5x10^6 splenocytes from B6 WT and IL-17C^-/-^ donors respectively. Serums were collected 14 dayspost-transplantation and cytokines production was examined by CBA; n=7-8 per group. Data are representative of at least three experiments and presented as mean ± SEM. **P* < 0.05, ***P*< 0.01.


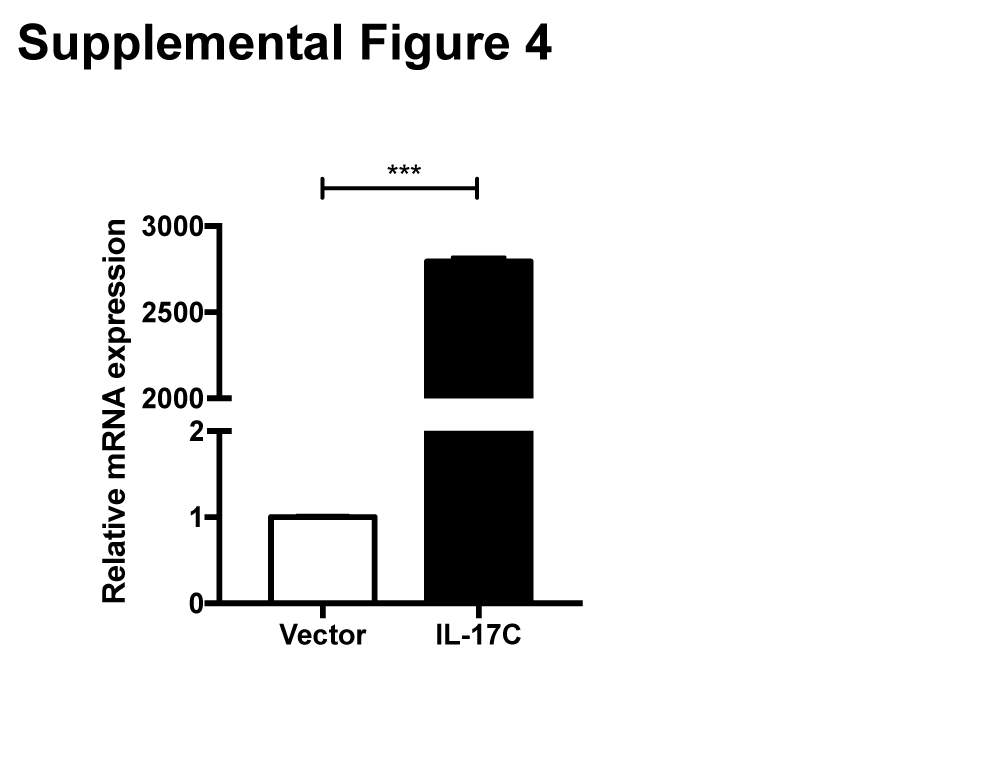


**Supplemental Figure 4.** IL-17C expression in recipients of minicircle-IL-17C plasmid and control plasmid injections. Vector plasmid or minicircle-IL-17C plasmid (60ug/mice) was injected into BALB/C recipients by hydrodynamic gene transfer (HGT). After 5 days, IL-17C expression level in the liver was assessed by real-time PCR. Data are representative of at least three experiments and presented as mean ± SEM. ****P*< 0.001.


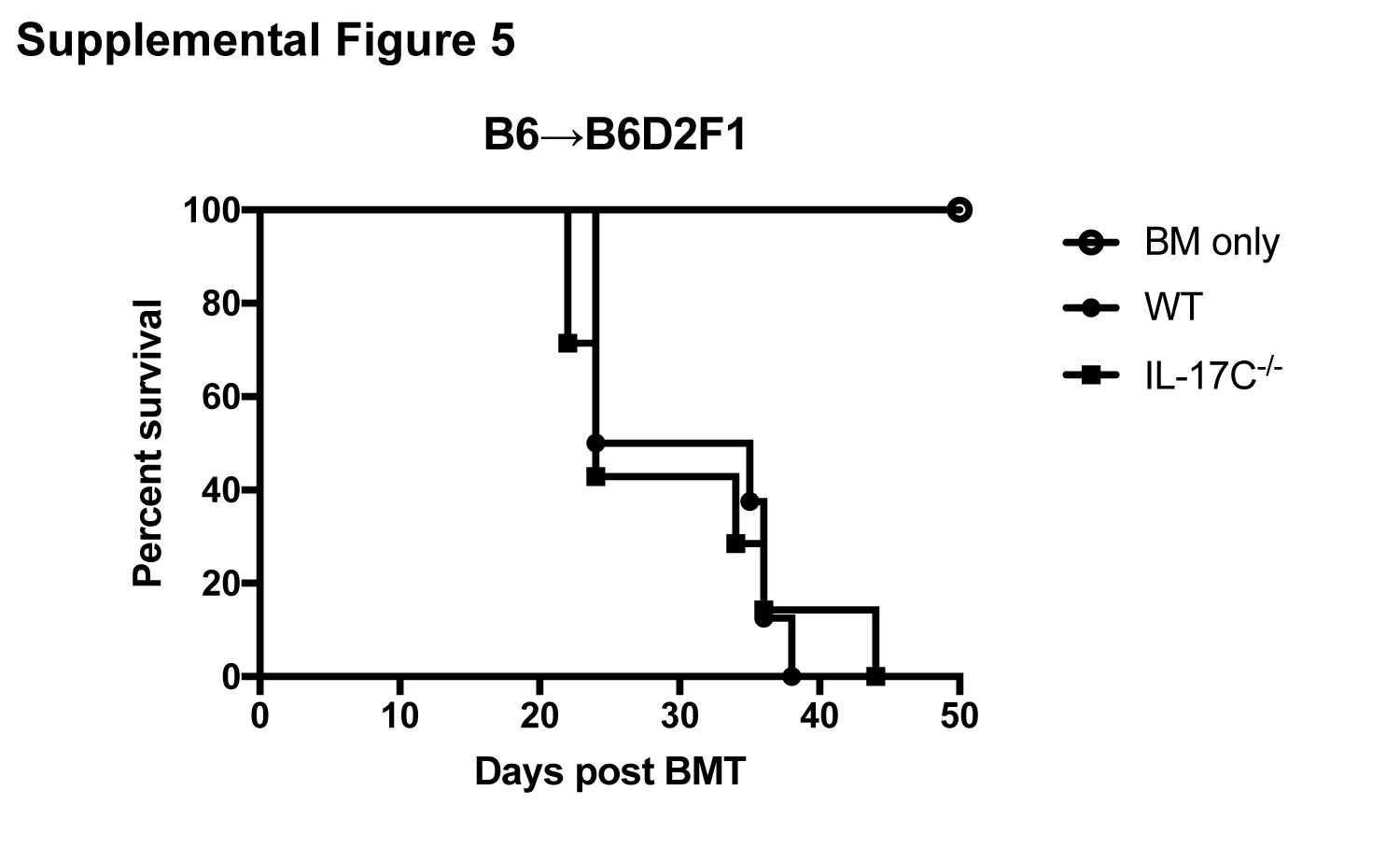


**Supplemental Figure 5.** IL-17C has no effect in haplo-identical aGVHD model. B6D2F1 mice were lethally irradiated and transplanted with 1x10^7 BM cells and 7.5x10^7 splenocytes from B6 WT or IL-17C^-/-^ donors respectively. Survival of the recipient mice was monitored. BM only, n=3; WT, n=7; IL-17C^-/-^, n=7. Data displayed is from one experiment.


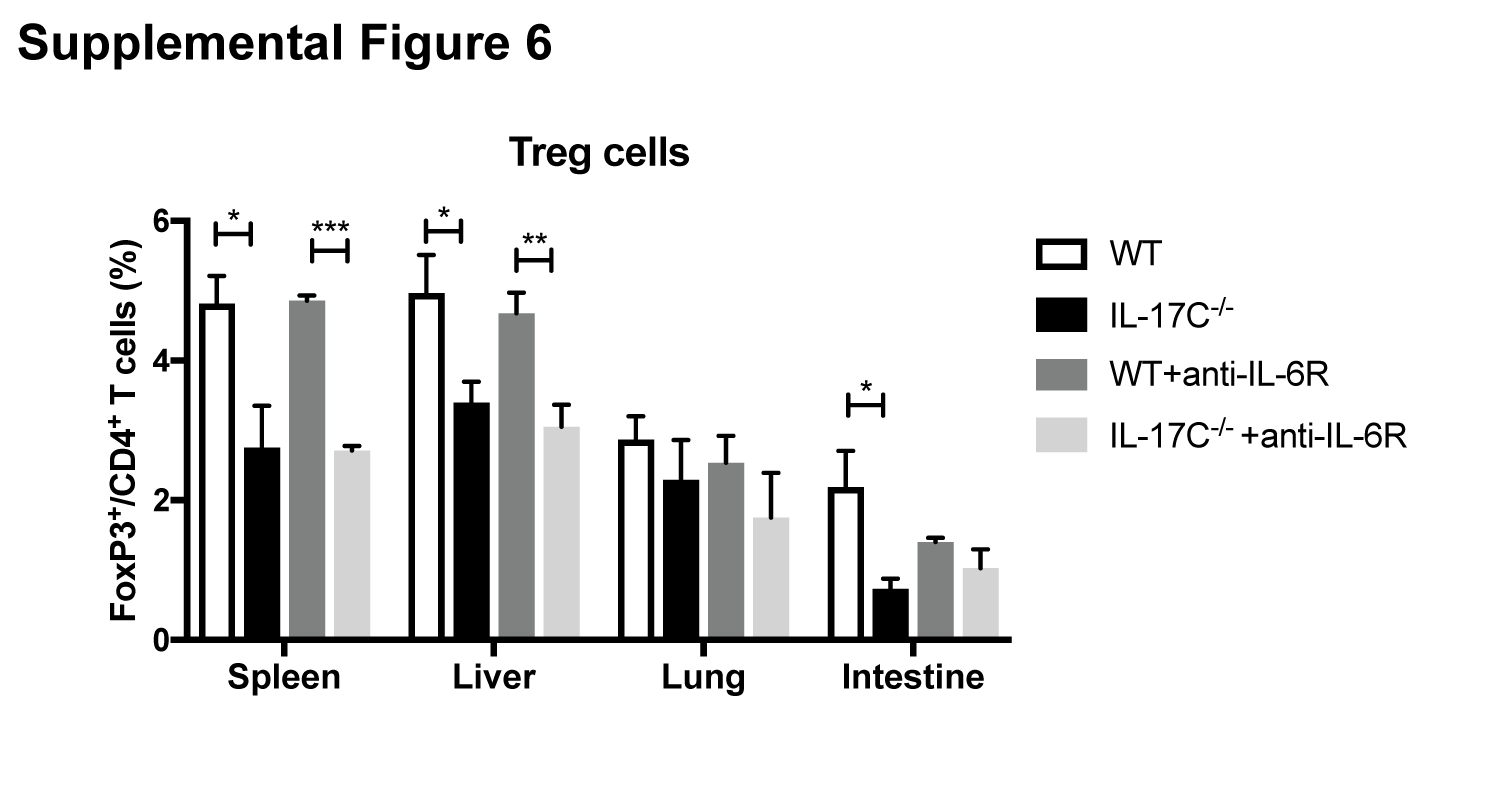


**Supplemental Figure 6.** IL-6 is dispensable for the increased Treg differentiation in recipients of IL-17C^-/-^ grafts. Lethally irradiated BALB/c recipients were transplanted with 1x10^7 BM cells and 5x10^6 splenocytes from B6 WT and IL-17C^-/-^ donors respectively. Recipients were intraperitoneally administrated anti-mouse IL-6R or rat IgG2b antibody on day -1, 3, and 7. Percentage of Treg cells was examined 14 days post transplantation; n=6-7 per group. Data are representative of at least three experiments and presented as mean ± SEM. **P* < 0.05, ***P*< 0.01, ****P*< 0.001.
